# Comparative Prognostic Value of Ion Shift Index and Naples Prognostic Score for Predicting In-Hospital Mortality in STEMI Patients: A Single-Center Retrospective Study

**DOI:** 10.3390/diagnostics15172186

**Published:** 2025-08-28

**Authors:** İbrahim Halil Yasak, Ramazan Giden, Esat Barut

**Affiliations:** 1 Department of Emergency Medicine, Faculty of Medicine, Harran University, Sanliurfa 63040, Turkey; dr.ramazangiden@gmail.com; 2 Dr. Yusuf Azizoglu State Hospital Silvan, Diyarbakır 21640, Turkey; esbarut94@gmail.com

**Keywords:** STEMI, Ion Shift Index, Naples Prognostic Score, in-hospital mortality, prognosis

## Abstract

**Background/Objectives:** Acute myocardial infarction with ST-segment elevation (STEMI) remains a clinical condition with high mortality. The Ion Shift Index (ISI) and Naples Prognostic Score (NPS) are two prognostic indicators that have recently come to the fore. The aim of this study is to compare the predictive value of ISI and NPS in predicting in-hospital mortality in STEMI patients. **Methods:** This retrospective study included 214 STEMI patients (1 January 2022–1 January 2024). Exclusion criteria included active cancer, infection, autoimmune disease, or chronic kidney disease. ISI and NPS were calculated from laboratory results obtained from the emergency department at the time of initial presentation. Patients were categorized according to in-hospital survival. Logistic regression and ROC curve analyses were performed for in-hospital mortality. **Results:** The mean age of participants was 64.8 ± 11.2 years, and 40.2% were female; a total of 36 patients (16.8%) died during hospitalization. Hypertension and female gender were more common in those who died, and LDL cholesterol and inflammatory markers were higher. The ISI value was significantly increased in the mortality group, whereas no significant difference was observed in NPS. ROC analysis revealed that at a threshold value of 3.0, ISI had a sensitivity of 68% and specificity of 71%, with an area under the curve (AUC) of 0.70, while NPS had an AUC of 0.55 and did not demonstrate significant discriminatory power. In the multivariate analysis, ISI and increased LDL cholesterol were independently associated with mortality; decreased lymphocyte/monocyte ratio and female gender were also additional independent predictors. NPS did not emerge as an independent factor in predicting in-hospital mortality. **Conclusions:** ISI was found to be a superior and independent early risk predictor of in-hospital mortality in STEMI patients compared to NPS. ISI may serve as a rapid and inexpensive risk classification tool in the acute phase, as it reflects sudden changes in intracellular–extracellular ion balance, whereas NPS may not be sufficiently sensitive in the hyperacute phase, as its components reflect chronic nutritional and inflammatory states. Due to limitations such as a single-center retrospective design and low mortality rates, validation through multicenter prospective studies is required for the integration of ISI into clinical practice.

## 1. Introduction

Acute coronary syndrome (ACS) is one of the most prevalent global public health problems [1]. ST-elevation myocardial infarction (STEMI), which accounts for approximately 30% of ACS cases, is associated with high morbidity and mortality rates [2]. If not treated promptly, it can lead to severe complications and represents a clinical emergency [3]. While inflammation plays a significant role in the pathogenesis of STEMI, electrolyte imbalances may also influence the prognosis of STEMI patients [4,5]. 

### 1.1. Pathophysiology of STEMI and Electrolyte Disturbances

The fundamental pathophysiology of STEMI involves myocardial ischemia due to the complete occlusion of the coronary artery lumen by a thrombus following atherosclerotic plaque rupture. This process results in decreased ATP production and dysfunction of energy-dependent ion pumps, causing intracellular accumulation of sodium and calcium, loss of potassium, and disruption of cardiac electrical activity. As a result, the risk of arrhythmia and cellular injury increases significantly [6].

Electrolyte disturbances during STEMI play a critical role in both acute complications and long-term prognosis. Alterations in potassium, sodium, calcium, and magnesium levels directly affect cardiac electrical stability and myocardial contractility. Notably, potassium levels—even within normal limits—have been reported as independent predictors of major in-hospital cardiac events [7]. Changes in sodium, calcium, and magnesium concentrations also significantly impact the course of the disease and cardiac function. Hyponatremia is associated with the severity of myocardial injury and poor prognosis [8]. Intracellular calcium overload impairs contractility, and both serum calcium levels and the calcium/albumin ratio have been linked to the extent of coronary artery disease [9]. Magnesium plays a crucial role in cardiac contraction and electrical conduction; its deficiency increases arrhythmia risk, especially in older adults [10].

### 1.2. Inflammatory Response in STEMI

The inflammatory response after myocardial infarction begins rapidly as a result of ischemia-induced cell death and necrosis [11]. Leukocyte count is an important biomarker in assessing the inflammatory response in STEMI. Leukocytosis at admission indicates systemic inflammation and is associated with disease severity and complication risk. In the study by Göçer and Gedik, significantly elevated levels of leukocytes, neutrophils, procalcitonin, and CRP were observed in STEMI patients. These findings suggest that the inflammatory response can be monitored using hematologic and biochemical parameters and may help in early prediction of complications [5].

### 1.3. Current Prognostic Scoring Systems

Significant progress has been made in recent years in risk stratification of STEMI patients. The TIMI score provides early prediction based on clinical variables such as age, diabetes, and hypertension, while the GRACE score offers a more comprehensive evaluation by incorporating hemodynamic and laboratory parameters [12]. The CRUSADE score is particularly valuable for predicting bleeding risk [13].

However, these traditional scoring systems are limited by computational complexity and their inability to fully capture all the pathophysiological processes specific to STEMI. Therefore, there is a growing need for newer prognostic tools that are simpler, faster, and better suited to clinical practice.

Recent studies have explored alternative indices such as the Naples Prognostic Score (NPS) and the Ion Shift Index (ISI) in the context of myocardial infarction. Wang et al. demonstrated that NPS at admission has prognostic value for long-term outcomes in patients with coronary artery disease, while Geltser et al. reviewed advances in risk assessment methods, highlighting the potential of NPS among other tools for predicting hospital mortality in STEMI patients [14,15].

### 1.4. Naples Prognostic Score: A Multiparametric Approach

The Naples Prognostic Score (NPS) is a multiparametric scoring system based on inflammation, nutritional status, and lipid metabolism [16]. Initially developed for use in oncology, it has also demonstrated prognostic value in cardiovascular diseases. This score incorporates biomarkers such as albumin, cholesterol, neutrophil-to-lymphocyte ratio (NLR), and lymphocyte-to-monocyte ratio (LMR), and has shown meaningful predictive value for ejection fraction and long-term mortality in STEMI patients [17,18,19,20].

### 1.5. Ion Shift Index: A Novel Electrolyte-Based Prognostic Tool

The Ion Shift Index (ISI) is a simple prognostic indicator reflecting electrolyte imbalance, based on serum levels of potassium, phosphate, magnesium, and calcium [21]. Originally developed to assess the degree of cellular injury in cardiac arrest and trauma patients, higher ISI values have been associated with more severe cellular damage and physiological instability [22,23,24]. Studies conducted in intensive care unit settings have demonstrated the utility of ISI in predicting mortality and neurological outcomes [25].

### 1.6. Study Objectives

This study aims to compare the prognostic power of ISI and NPS in predicting in-hospital mortality in STEMI patients.

## 2. Materials and Methods

### 2.1. Study Design and Study Population

This study was conducted using a retrospective and observational design between January 2022 and January 2024 in the emergency department of a tertiary university hospital in Turkey. Ethical approval was obtained from the Ethics Committee of Harran University (Ethics Committee Approval No: HRU/24.11.31), and the study was conducted in accordance with the principles of the Declaration of Helsinki.

A total of 214 patients aged 18 years and older who were diagnosed with ST-segment elevation myocardial infarction (STEMI) upon presentation were included in the study. The diagnosis of STEMI was based on elevated cardiac troponin levels in combination with chest pain lasting longer than 20 min and ST-segment elevation of ≥0.1 mV in at least two contiguous leads other than V2–V3, or ≥0.2 mV in men aged ≥40 years, ≥0.25 mV in men <40 years, or ≥0.15 mV in women in V2–V3 leads on electrocardiography [1].

### 2.2. Inclusion and Exclusion Criteria

The inclusion criteria were as follows:

- A confirmed diagnosis of STEMI at presentation;
- Availability of laboratory data within the first two hours following intervention;
- Accessible information regarding in-hospital outcomes.

The exclusion criteria included the following:

- Presence of active infection, sepsis, or autoimmune disease;
- History of malignancy or known chronic liver disease;
- End-stage renal disease or current dialysis treatment;
- Transfer to another medical center before hospital admission was completed.

These exclusion criteria were defined to eliminate potential confounding effects caused by conditions that could independently affect inflammatory response, nutritional status, or electrolyte balance apart from myocardial infarction.

### 2.3. Data Collection and Laboratory Findings

Demographic, clinical, and laboratory data were retrospectively retrieved from the hospital’s digital records system. Recorded variables included age, sex, comorbidities (diabetes mellitus, hypertension, hyperlipidemia), vital signs, ECG findings, echocardiographic ejection fraction, and in-hospital mortality status. Echocardiographic ejection fraction, assessed within the first 24 h of hospital admission.

Laboratory parameters evaluated included the following:

- Hematological tests: Complete blood count (white blood cell count, neutrophils, lymphocytes, monocytes, hemoglobin);
- Biochemical tests: Serum glucose, creatinine, total cholesterol, albumin, LDL cholesterol;
- Electrolytes: Potassium (K^+^), calcium (Ca^2+^), phosphate (PO_4_^3−^), magnesium (Mg^2+^).

All blood samples were collected within the first two hours after presentation and analyzed under standardized conditions using automated analyzers in the hospital’s central laboratory. This timing was deliberately chosen to reflect the acute phase of STEMI.

### 2.4. Calculation of ISI and NPSs

ISI was calculated using the following formula [20]:

ISI (arbitrary unit) = (Potassium [mmol/L] + Phosphate [mmol/L] + Magnesium [mmol/L])/Calcium [mmol/L].

All parameters were evaluated in mmol/L.

The Naples Prognostic Score (NPS) was calculated according to the method defined by Galizia et al. [16]. Four criteria were assessed, each contributing one point if present (Table 1). Patients were divided into three risk groups based on their total score: low risk (0), moderate risk (1–2), and high risk (3–4) (Table 2).

### 2.5. Outcomes Assessed

The primary endpoint of the study was all-cause in-hospital mortality among patients admitted for STEMI. Secondary outcomes included the associations of ISI and NPS with demographic and laboratory parameters.

### 2.6. Statistical Analysis

Statistical analyses were performed using IBM SPSS Statistics version 26.0 (IBM Corp., Armonk, NY, USA). Continuous variables were presented as mean ± standard deviation (SD) or median (interquartile range, IQR), depending on the distribution. Normality was assessed using the Shapiro–Wilk test. Categorical variables were expressed as counts and percentages (%).

The following statistical tests were used to compare survivors and non-survivors:

- For continuous variables: Independent samples *t*-test or Mann–Whitney U test;
- For categorical variables: Chi-square test or Fisher’s exact test.

Univariate logistic regression analyses were first performed to screen potential predictors of in-hospital mortality. Variables with a *p*-value < 0.10 in these univariate analyses were subsequently entered into a multivariate logistic regression model to identify independent predictors. Results are presented as odds ratios (OR) with 95% confidence intervals (CI). A *p*-value < 0.05 was considered statistically significant.

## 3. Results

### 3.1. Basic Clinical Characteristics

Data from 214 patients included in the study and diagnosed with STEMI were analyzed. The overall in-hospital mortality rate was 16.8% (*n* = 36). The mean age of the participants was 64.8 ± 11.2 years, and female patients accounted for 40.19% of the total group.

When comparing discharged and deceased patients, no statistically significant difference was observed in terms of age. However, female gender was significantly more prevalent in the mortality group. In addition, the prevalence of hypertension was significantly higher among those who died (Table 3).

### 3.2. Laboratory Data

LDL cholesterol levels were found to be significantly higher in patients who died during their hospital stay than in survivors. Similarly, significant differences were observed in inflammatory markers (Table 4).

Hemoglobin levels were similar between the discharge and mortality groups and were not associated with in-hospital death.

### 3.3. Comparison of the Ion Shift Index and the Naples Prognostic Score

ISI value was significantly higher in the group with mortality compared to survivors. In contrast, no significant difference was observed between the groups in terms of the NPS (Table 3).

The emergence of ISI as a more successful prognostic marker may be attributed to its ability to reflect acute cellular and metabolic impairments in real time. NPS, on the other hand, may not achieve sufficient sensitivity in the early stages because it is based on biochemical markers such as albumin and cholesterol, which vary over time.

**Figure 1:**
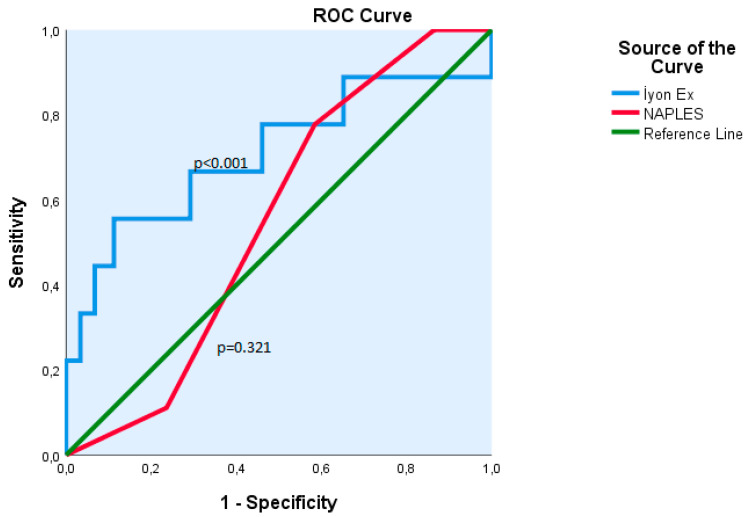
ROC curves of the ISI and NPS for predicting mortality. For ISI AUC = 0.70 and NPS AUC = 0.55.

Receiver operating characteristic (ROC) curve analysis demonstrated that the ISI, at an optimal cut-off value of 3.0, achieved a sensitivity of 68% and a specificity of 71%, with statistical significance (*p* < 0.001). The area under the curve (AUC) for ISI was 0.70, and the AUC value for NPS was 0.55. NPS did not show statistically significant discriminatory ability (*p* = 0.321). The comparative ROC curves for both parameters are illustrated in Figure 1.

### 3.4. Correlation and Regression Findings

In univariate logistic regression analysis, the ISI, LDL cholesterol, gender, hypertension, glucose, ejection fraction and LMR were associated with in hospital mortality at the *p* < 0.10 level (Table 5). The NLR was not significant (*p* = 0.127) and was therefore not included in the multivariate model.

These variables were subsequently entered into a multivariate logistic regression mod-el. In the multivariate analysis, ISI, LDL cholesterol, gender and LMR remained independent predictors of mortality, whereas hypertension, glucose and ejection fraction lost significance after adjustment.

### 3.5. Subgroup Analysis by Gender

In the present study, a significant difference in the ISI was observed between sexes. The median ISI was 3.02 in females and 2.77 in males, with this difference reaching statistical significance. In contrast, there was no significant difference in the NAPLES score between females and males (Table 6).

## 4. Discussion

This study aimed to comparatively evaluate the prognostic value of the ISI and NPS systems in predicting in-hospital mortality in patients presenting to the emergency department with a diagnosis of STEMI. The findings revealed that ISI is a significant and independent predictor of early mortality, whereas NPS did not show statistical significance in this context. These results highlight the potential of ISI as a real-time risk classification tool in acute coronary syndromes.

Despite advances in diagnosis and treatment, STEMI remains a leading cause of death worldwide [1].

### 4.1. The Role of ISI in Determining Acute Mortality

In our study, we found that ISI values were significantly higher in patients who died during hospitalization, and that ISI remained an independent predictor of death in multivariate regression analysis. These findings are consistent with previous studies conducted in different patient groups. ISI was first defined by Lee and colleagues in cardiac arrest patients and demonstrated its ability to predict neurological outcomes and survival [21]. Subsequent studies in trauma, surgical, and intensive care patients confirmed that elevated ISI values are associated with increased mortality [22,23,24].

The physiological basis of ISI is based on its direct reflection of acute cellular damage and metabolic crisis [22]. During ischemia, the loss of function of ion pumps causes potassium, magnesium, and phosphate to leak from damaged cells into the external environment, while calcium floods into the cells [21]. These ion changes disrupt the stability of the cell membrane, predispose to arrhythmias, and are proportional to the severity of the damage. In this respect, ISI functions as an indicator of immediate metabolic stress, unlike classic inflammatory markers, which lag behind clinical manifestations [23].

In our study, ISI remained a significant predictor even after adjusting for potential confounding factors such as age, gender, hypertension, LDL cholesterol, and albumin, indicating that this index directly reflects acute myocardial damage.

To the best of our knowledge, this is the first study to evaluate and validate the use of ISI in patients with STEMI. These findings highlight the potential for broader application of ISI across the spectrum of critical illness.

### 4.2. Limitations of the Naples Prognostic Score in Acute Clinical Conditions

The NPS is a score developed to assess the long-term prognosis of patients undergoing gastrointestinal cancer surgery [16]. This system is based on components that reflect nutritional status and inflammatory response. In the cardiovascular field, many studies have shown that NPS has prognostic value. For example, Erdoğan et al. reported that higher NPS values at hospital admission were associated with increased in-hospital mortality and left ventricular dysfunction in STEMI patients undergoing primary PCI [17]. Similarly, Saygı et al. found that NPS independently predicted both short-term and one-year mortality in a multicenter STEMI cohort [20]. Birdal et al. also demonstrated a significant correlation between high NPS after STEMI and deterioration in left ventricular ejection fraction [19]. These studies suggest that NPS can be used as a valuable predictor of adverse outcomes in patients with STEMI.

However, in our study, NPS was not significant in predicting in-hospital mortality rates. This can be explained by the nature of the components that make up the score. Parameters such as serum albumin and cholesterol levels reflect chronic inflammation and nutritional status rather than the rapid physiological changes that occur in the hyperacute phase of STEMI. Changes in these biomarkers typically take days to become evident. Therefore, while NPS appears to be a valuable tool for assessing subacute and long-term risk in cardiovascular patients, its sensitivity in detecting early-stage mortality risk in STEMI appears to be limited.

Our findings indicate that, among the inflammatory components of the NPS, the LMR is the only parameter significantly associated with in hospital mortality, and it remains an independent predictor in multivariate analysis. By contrast, the NLR did not achieve statistical significance in our cohort and was therefore omitted from the final regression model. Similarly, variables reflecting nutritional status—namely se-rum total cholesterol and albumin concentrations—were not related to early mortality [26].

This pattern suggests that the prognostic contribution of the NPS in the hypera-cute phase of STEMI may derive mainly from the LMR component, whereas its other components (NLR, total cholesterol and albumin) do not carry predictive value in this context. Combining heterogeneous biomarkers that reflect different physiological pro-cesses—some chronic (nutritional status) and some acute (inflammatory re-sponse)—into a single score may therefore dilute the discriminatory power of the NPS. For acute prognostication, indices that directly capture immediate metabolic stress, such as the ISI, appear to be more sensitive.

### 4.3. Comparison with Traditional Risk Scores

Traditional cardiovascular risk assessment relies heavily on established scoring methods such as TIMI, GRACE, and CRUSADE frameworks, which combine various clinical parameters, hemodynamic measurements, and laboratory findings to measure the likelihood of death [12,13]. The TIMI risk classification tool, originally formulated for acute coronary syndromes without initial ST elevation, has since demonstrated its applicability in STEMI cohorts and shown reasonable discriminatory capacity for early mortality outcomes [27]. In contrast, the GRACE risk algorithm has consistently outperformed alternative models. Comprehensive international registry data document its superior predictive performance for both acute in-hospital mortality and six-month mortality outcomes in STEMI cases [12].

However, these prognostic tools present practical challenges in acute care settings. Both the TIMI and GRACE methodologies require comprehensive data collection encompassing numerous variables. Many of these variables, such as hemodynamic classification systems, resuscitation events during admission, or the creation of a comprehensive metabolic profile, may not be obtainable during the initial evaluation in the emergency department. Additionally, these scoring systems cannot quickly identify acute cellular dysfunction or metabolic disorders that can be assessed rapidly through parameters derived from electrolytes, such as the Ion Shift Index.

In this clinical context, the ISI offers significant operational benefits: rapid calculation from standard electrolyte measurements obtained in the first minutes of the patient’s presentation, facilitating rapid clinical decision-making and risk categorization. While the TIMI and GRACE methodologies maintain their position as reference standards for comprehensive outcome prediction, our research demonstrates that ISI can function as a complementary diagnostic tool and may be particularly useful in acute care scenarios where urgent clinical decisions must be made before all traditional scoring parameters are available.

### 4.4. Gender-Related Differences in ISI and Prognosis

A large single-center study showed that patients with high serum potassium levels were older and predominantly female, and that high serum potassium levels increased the risk of major cardiovascular events by approximately 20% and the risk of death from all causes by more than 70% [28].

The study, titled Medical Information Mart for Intensive Care, revealed that women who suffer from acute myocardial infarction have a higher one-year mortality risk than men [29]. In our gender subgroup analysis, consistent with the literature, we found that female patients had significantly higher ISI values than males, but no significant difference in NPS values. These data suggest that electrolyte imbalance may play a role in gender-related prognostic differences.

### 4.5. Relationship Between LDL Cholesterol and Mortality

In our study, LDL cholesterol levels were also identified as an independent predictor of mortality. The association between elevated LDL levels and adverse clinical outcomes has previously been described as a marker of atherosclerotic burden and plaque instability [30]. Conversely, some studies have reported a link between low LDL levels and increased mortality; however, this inverse relationship is often attributed to the presence of chronic illness or non-causal associations [31].

Lipid metabolism plays a complex role in regulating inflammatory processes, endothelial function, and immune responses [32]. Oxidized LDL particles can trigger foam cell formation and plaque rupture. In this context, elevated LDL levels at admission may reflect uncontrolled atherosclerosis and increased cardiovascular risk [33]. This underscores the necessity of integrating LDL into risk prediction models.

Recent cohort studies have reported that low LDL and total cholesterol levels are associated with higher mortality, a phenomenon referred to as the “cholesterol paradox.” A long-term case–control study conducted in Sweden revealed that low LDL-C and total cholesterol levels are associated with increased mortality, while a large acute coronary syndrome cohort in China showed that LDL-C levels <70 mg/dL independently adversely affect long-term survival and are associated with higher CRP levels [34,35].

These findings suggest that low LDL in acute myocardial infarction may not be protective but rather a marker of inflammation, malnutrition, or acute-phase response.

### 4.6. Clinical Applications and Future Perspectives

The data obtained show that ISI can be an effective tool for the early identification of high-risk patients diagnosed with STEMI in the emergency department. Its ease of calculation and reliance on common laboratory parameters make it possible to integrate this score into clinical workflows. Especially as an alternative to complex and time-consuming scoring systems, ISI can provide faster and more effective assessment in certain clinical situations.

Future studies should elucidate the potential contribution of ISI when used in conjunction with established scores such as GRACE or TIMI. Additionally, the predictive power of ISI in post-hospitalization periods has not yet been clarified. Therefore, prospective, multicenter studies and external validation studies are needed to determine standardized cutoff values for ISI and confirm its generalizability.

Furthermore, the impact of ISI-based clinical interventions—such as more aggressive reperfusion strategies, electrolyte balance, or intensive care monitoring—on patient outcomes should be investigated; thus, ISI could become not only a prognostic tool but also a target that can guide treatment.

This study has several limitations. Most importantly, it was conducted retrospectively at a single tertiary center, which inherently limits the generalizability of the results to broader and more diverse STEMI populations. The single-center design may also reflect institutional practices and regional patient characteristics that differ from other settings. Only in-hospital mortality was assessed, without evaluation of long-term outcomes. ISI and NPS were calculated based on initial lab values only, not reflecting dynamic changes over time. Lastly, due to retrospective design and limitations in the hospital’s electronic medical record system, detailed angiographic data such as infarction location, number of affected coronary arteries, treatment method, and revascularization could not be obtained for all patients, which may have affected prognostic clinical variables in STEMI.

The relatively low number of deaths in the study (*n* = 36) limited the statistical power of the multivariate logistic regression analysis. The event-variable ratio was approximately 5 (36 events divided by seven predictors), which increased the risk of overfitting. Although recent simulation studies have shown that the rule of 10 events per variable may be overly conservative, caution should be exercised in interpreting the regression findings.

## 5. Conclusions

This study demonstrated that the ISI is an independent and strong predictor of in-hospital mortality in patients with STEMI. By reflecting acute shifts in electrolytes at the cellular level, ISI offers significant value for early risk stratification in clinical settings. In contrast, the NPS did not show a meaningful association with early mortality in this cohort.

Moreover, the association between low calcium and albumin levels and elevated glucose, LDL, and NLR levels with mortality underscores the critical role of acute-phase inflammation and metabolic imbalance in determining outcomes in STEMI. The simplicity and low cost of ISI—derived from routine laboratory parameters—make it an attractive tool for rapid risk assessment in emergency and intensive care settings.

Future multicenter, prospective studies are needed to further validate the prognostic utility of ISI and to explore its integration into standard risk scoring systems. Such efforts could enhance the early identification of high-risk STEMI patients who may benefit from more aggressive monitoring and therapeutic intervention.

## Figures and Tables

**Table 1:** Calculation of Naples Score.

*Variables*	Values	Score
*Total Cholesterol (mg/dL)*	>180	0
	≤180	1
*Albumin (g/L)*	≥40	0
	<40	1
*LMR*	>4.44	0
	≤4.44	1
*NLR*	≤2.96	0
	>2.96	1

LMR: lymphocyte-to-monocyte ratio; NLR: neutrophil-to-lymphocyte ratio.

**Table 2:** Risk Groups Based on Nagles Score Points.

*Categorize*	*Score*
*Low risk*	0
*Intermediate risk*	1–2
*High risk*	3–4

**Table 3:** Baseline demographic characteristics and risk factors of STEMI patients classified as discharged or deceased according to in-hospital outcomes.

* Variables *	Discharged (*n*: 178)	Deceased (*n*: 36)	*p*
*Age*	65 ± 10.5	64 ± 3.3	0.776
*Gender (Female)*	58 (32.6%)	28 (77.7%)	<0.001
*Diabetes Mellitus (%)*	56 (31.5%)	16 (44.4%)	0.175
*Hypertension (%)*	68 (38.2%)	24 (66.7%)	0.003

**Table 4:** Laboratory and echocardiographic variables of discharged and deceased patients.

* Variables *	Discharged (*n*: 178)	Deceased (*n*: 36)	*p*
*Glucose (mg/dL)*	135 (114–199)	291 (179–444)	<0.001
*Creatinine (mg/dL)*	0.84 ± 0.22	0.9 ± 0.11	0.099
*WBC (10 × 10^3^/uL)*	11.74 ± 4.28	10.58 ± 2.79	0.122
*Hemoglobin (g/dL)*	12.64 ± 1.92	12.61 ± 1.92	0.937
*Potassium (mmol/L)*	4.43 ± 0.70	4.44 ± 0.99	0.959
*Calcium (mg/dL)*	2.16 ± 0.19	2.00 ± 0.15	<0.001
*Phosphorus (mg/dL)*	1.04 ± 0.31	1.88 ± 1.15	<0.001
*Magnesium (mg/dL)*	0.78 ± 0.13	0.87 ± 0.18	0.001
*Albumin (g/dL)*	4.00 ± 0.47	3.39 ± 0.69	<0.001
*Cholesterol (mg/dL)*	163.72 ± 41.71	160.89 ± 33.99	0.702
*Triglycerides (mg/dL)*	161 ± 103	132 ± 50.4	0.003
*HDL (mg/dL)*	37.23 ± 12.58	41.74 ± 15.04	0.059
*LDL (mg/dL)*	102.66 ± 37.55	115.47 ± 7.86	0.043
*NLR*	7.36 ± 6.25	10.05± 10.48	0.041
*LMR*	3.62 ± 2.16	4.95 ± 3.85	0.004
*Troponin (pg/mL)*	964.9 (109–19,417)	1183.0 (260–8659)	0.789
*Ejection Fraction (%)*	43.87 ± 6.44	46.17 ± 9.15	0.172
*Naples Score*	2.64 ± 1.08	2.89 ± 0.58	0.180
*Ion Shift Index (mmol/L)*	2.92 ± 0.56	3.64 ± 1.21	<0.001

WBC: white blood cell count; Hb: hemoglobin; K: potassium; Ca: calcium; P: phosphorus; Mg: magnesium; TG: triglycerides; HDL: high-density lipoprotein; LDL: low-density lipoprotein; NLR: neutrophil-to-lymphocyte ratio; LMR: lymphocyte-to-monocyte ratio; EF: ejection fraction.

**Table 5:** Univariate and multivariate logistic regression models assessing predictors of in-hospital mortality in STEMI patients.

* Variables *	Univariate OR (95% CI)	*p*	Multivariate OR (95% CI)	*p*
*Ion Shift Index*	2.779 (1.794–4.303)	<0.001	2.52 (1.533–4.148)	<0.001
*Glucose*	0.995 (0.990–1.001)	0.087	0.996 (0.990–1.002)	0.230
*LDL*	1.010 (1.000–1.020)	0.047	1.014 (1.002–1.026)	0.023
*LMR*	0.181 (0.018–0.344)	0.03	0.254 (0.082–0.426)	0.004
*Ejection Fraction*	1.050 (0.995–1.109)	0.074	1.019 (0.962–1.079)	0.530
*Gender*	7.241 (3.108–16.874)	<0.001	0.199 (0.082–0.484)	<0.001
*Hypertension*	0.309 (0.145–0.658)	0.002	0.514 (0.215–1.227)	0.134
*NLR*	0.041 (0.012–0.094)	0.127	-	-
*Troponin*	0.908 (0.806–1.020)	0.112	-	-
*Age*	0.995 (0.958–1.032)	0.775	-	-
*Naples Score*	1.297 (0.886–1.897)	0.181	-	-

LDL: low-density lipoprotein; LMR: lymphocyte-to-monocyte ratio; NLR: neutrophil-to-lymphocyte ratio.

**Table 6:** Comparison of the ISI and NAPLES score according to sex.

* Variables *	Female	Male	*p*-Value
***ISI***	3.02 ± 1.05	2.77 ± 0.61	<0.001
***NPS***	3 ± 0.932	3 ± 1.02	0.335

## Data Availability

The data supporting the findings of this study are available from the corresponding author upon reasonable request.

## Abbreviations

STEMI	ST-Elevation Myocardial Infarction
ISI	Ion Shift Index
NPS	Naples Prognostic Score
LDL	Low-Density Lipoprotein
NLR	Neutrophil-to-Lymphocyte Ratio
LMR	Lymphocyte-to-Monocyte Ratio
SPSS	Statistical Package for the Social Sciences
OR	Odds RatioCI—Confidence Interval
